# Nutritional Interventions May Improve Outcomes of Patients Operated on for Diabetic Foot Infections: A Single-Center Case-Control Study

**DOI:** 10.1155/2022/9546144

**Published:** 2022-08-05

**Authors:** Ilker Uçkay, Vinoth Yogarasa, Felix W. A. Waibel, Annette Seiler-Bänziger, Maja Kuhn, Margrit Sahli, Martin C. Berli, Benjamin A. Lipsky, Madlaina Schöni

**Affiliations:** ^1^Infectiology, Balgrist University Hospital, University of Zurich, Switzerland; ^2^Diabetic Foot Unit, Department of Orthopedic Surgery, Balgrist University Hospital, University of Zurich, Switzerland; ^3^Nutritionist Service, Balgrist University Hospital, University of Zurich, Switzerland; ^4^Department of Medicine, University of Washington, Seattle, USA

## Abstract

**Aim:**

While a patient's nutritional status is known to generally have a role in postoperative wound healing, there is little information on its role as therapy in the multifaceted problem of diabetic foot infections (DFIs).

**Methods:**

We assessed this issue by conducting a retrospective case-control cohort study using a multivariate Cox regression model. The nutrition status of the DFI patients was assessed by professional nutritionists, who also orchestrated the nutritional intervention (counselling, composition of the intrahospital food) during hospitalization.

**Results:**

Among 1,013 DFI episodes in 586 patients (median age 67 years; 882 with osteomyelitis), 191 (19%) received a professional assessment of their nutrition accompanied by between 1 and 6 nutritional interventions. DFI cases who had professional nutritionists' interventions had a significantly shorter hospital stay, had shorter antibiotic therapies, and tended to fewer surgical debridements. By multivariate analysis, episodes with low Nutritional Risk Status- (NRS-) Scores 1-3 were associated with significantly lower failure rates after therapy for DFI (Cox regression analysis; hazard ratio 0.2, 95% confidence interval 0.1-0.7).

**Conclusions:**

In this retrospective cohort study, DFI episodes with low NRS-Score were associated with lower rates of clinical failure after DFI treatment, while nutritional interventions improved the outcome of DFI. We need prospective interventional trials for this treatment, and these are underway.

## 1. Introduction

Persons with diabetes are prone to a variety of foot complications such as limb ischemia, peripheral neuropathy, and infections [1–3]. Worldwide, the number of diabetic foot infections (DFIs) is increasing exponentially [1, 2]. Several publications have found an inverse association between relevant signs of malnutrition and the capacity to heal diabetic foot ulcers (DFUs) [4, 5]. These publications suggest there is a positive correlation between DFU healing and selected nutritional interventions [4, 5]. Likewise, our clinical experience suggests a worse outcome for many diabetic foot problems in patients with malnutrition and better outcomes when nutritional interventions are provided, at least for the postoperative period. Nevertheless, most clinicians are either ignorant of or largely ignore the associations between nutrition and outcomes after DFI treatment. This may be especially true for the many patients who undergo surgical debridement(s) and receive long-term systemic antibiotic therapy for DFI.

We investigated the potential associations of patient malnutrition at the time of presentation, as well as the results of consecutive nutritional interventions, on clinical outcomes. We were especially interested in associated variables, such as length of hospital stay, the number of surgical debridement, and the duration of antibiotic prescription. In this paper, we will not analyze the impact of professional diabetes counselling, as it is the subject of another upcoming publication.

## 2. Methods

We performed a single-center, case-control study examining the association of nutritional variables to four objective outcome parameters assessed during or after the combined surgical and medical management of DFI: clinical failure, length of hospital stay, number of surgical debridement, and total duration of antibiotic therapy. We defined DFI, including diabetic foot osteomyelitis (DFO), according to the criteria of the International Working Group on the Diabetic Foot (IWGDF) [1]. We defined “clinical failure” of DFI treatment as either a persistent, recurrent, or new relevant clinical problem at the original site occurring within one year of treatment. We defined “microbiological recurrence” as a clinically recurrent DFI at the same localization with cultures showing at least 50% of the same pathogen(s) as isolated in the index episode. This study belongs to our “DF-MANAG studies” to improve the management of DFIs and is approved by our Ethical Committee (BASEC 2019-01994).

We used a DFI registry that we developed, which lists all episodes occurring since the year 2000 [6]. One author (VT) completed the registry for his master degree, with five additional variables: an internationally validated nutrition score [7], a history of involuntary weight loss, treatment with vitamin substitutions or other nutritional interventions by our professional nutritionists, and the serum albumin level on admission (if available). Since 2003, we used the standardized NRS-Score (Nutritional Risk Screening Score 2002, with a range of 0 to 7) [7] throughout the study period. The NRS-Score screens the patient's nutritional status, the severity of malnutrition, and the age of the patient. The nutritional status is based on nutrition-related information acquired from patient interviews or the patient's weight or body mass index (BMI). The scoring system for severity of disease was created based on how well selected patient outcomes with certain diagnoses improved as a response to meeting daily caloric and protein requirements with nutritional support [7]. We purposely did not define the term “malnutrition,” since it is a confusing, complex term with no universally agreed definition. It may include phenomena related to starvation, and it can include patients who are overweight as well as those with low nutritional intake [8].

### 2.1. Nutritional Interventions

Our teaching hospital employs three nutritionists (220% full-time equivalence) who have a duration of professional experience in the field of 13, 32, and 35 years, respectively. Their first nutritional consultation usually takes 30 minutes, and follow-up visits generally last 10 to 30 minutes. Their main content is assessing the need for supplementation of proteins, especially for patients with nonhealing wounds. Other areas they assess are the choice of the optimal food for each individual's situation, the substitution of carbohydrates, and a general effort to encourage appropriate eating behaviors. These interventions are generally undertaken once to twice a week. Hence, the total number of interventions per DFI episode depends on the length of hospital stay, which is largely dictated by any postsurgical wound problems. The financial cost of an outpatient intervention is between 64 and 99 Swiss Francs (US$ 70-107). Inpatient consultations are part of the “diagnostic-related group” costs.

### 2.2. Statistical Analyses

Our primary outcome of interest was whether “clinical failure” was related to NSR-Scores on admission and whether professional nutritional counselling reduced the risk of clinical failure during, or after, therapy for DFI and after one year's follow-up. The secondary outcomes were the relationship to professional nutritional interventions of “microbiological recurrences,” length of hospital stay, number of surgical debridement, and the prescribed duration of antibiotic therapy. We compared groups using the Pearson *χ*^2^ or the Wilcoxon rank sum test. To adjust for the broad case-mix, we performed a multivariate Cox regression analysis for the primary outcome. With over 1,000 DFI episodes, the study was statistically well powered.

In the multivariate model, we intentionally omitted the variables “serum albumin” and “number of nutritional interventions,” because the former greatly interacts with the inflammation level [8, 9] and the latter is mostly determined by the duration of hospitalization (rather than the nutritional status). The secondary outcomes either were too few for analysis with a multivariate model or revealed substantial clinical interactions. Similarly, because of the arbitrary indication for nutritionists' consultations, i.e., mostly based on the treating clinicians' judgement, we elected against propensity score matching on the variable “nutritional intervention.” We used STATA™ software (Version 15; College Station, Texas, USA) and considered *p* values ≤ 0.05 (two-tailed) as statistically significant.

## 3. Results

### 3.1. Study Population and Infections

Among 1,013 DFI episodes in 586 patients (78% males; median age 67 years) occurring during the study period, 882 (87%) had DFO, 388 (38%) had a disorder associated with enhanced immunosuppression (beyond their diabetes mellitus) [6], and 753 (74%) were insulin-treated diabetes cases. At presentation, the patients' overall median duration of diabetes was 19 years. The median number of surgical interventions performed per DFI episode was 1; among them, 572 (56%) were some form of angioplasty of the affected limb. Culture results revealed 96 different bacterial constellations, which were treated with 46 different targeted antimicrobial regimens. The median duration of systemic antibiotic therapy was 21 days, of which 4 days were given intravenously. No patient was treated with any local antiseptic or antibiotic agents, bone substitutes, or hyperbaric oxygen. The median duration of medical follow-up for this cohort was 7.7 years.

### 3.2. Nutrition-Related Parameters

Among the 1,013 DFI episodes, the median for patients' weight was 87 kilograms, for mass 29 kg/m^2^, and 243 reported regular alcohol consumption. In a total of 191 episodes (19%), the patient received a nutritional intervention; the indicated reason was clinical “malnutrition” related to wound problems (93%) or involuntary weight loss (7%). Among the episodes with nutritional counselling, the median score on admission was 3 points. The distribution of the NRS-Scores was 0 in 6 cases (0.6%), 1 in 24 cases (2.4%), 2 in 19 cases (1.9%), 3 in 25 cases (2.5%), 4 in 13 cases (1.3%), and 5 in 14 cases (1.4%). All nutritional interventions targeted general protein and caloric substitution and provided tailored individual meals during hospitalization. Among the patients accepting the counselling, the median number of interventions was two (range, 1-6 per episode), and the distribution was single counselling (86/191 episodes, 47%), twice (54 times, 28%), three times (35 cases, 18%), four times (10 episodes, 5%), five times (1 episode, 1%), and six times (5 cases, 3%). Additional vitamin supplementation (not necessarily accompanied by professional counselling) was ordered or provided by clinicians, families, or the patients themselves in 251 (25%) cases.

### 3.3. Study Outcomes

Clinical failures were noted in 255 episodes (25%). Microbiologically proven recurrence of the index infection was noted in only 47 episodes (5% of the study population; 18% of the failures) [6].  Table 1 demonstrates a comparison of episodes of clinical failure with those of remission, including for nutrition-related parameters. We found no significant differences among the nutrition variables in episodes with versus without clinical failure (49/255 (19%) vs. 142/758 (24%); *p* = 0.87). In contrast, and after the case-mix adjustment by multivariate analysis, lower NRS-Scores (1-3 points) were associated with a significantly lower rate of therapeutic failures (Table 2). The receiver operating curve (ROC) value for this association was 0.75 (95% confidence interval 0.64-0.85), demonstrating good accuracy of our final statistical model. Regarding the secondary outcome, a nutritional intervention was associated with a significantly shorter hospital stay, a significantly shorter duration of (postsurgical) antibiotic therapy (Table 3), and a tendency to fewer surgical interventions.

## 4. Discussion

In this single-center study population of 1,013 adult patients treated with surgical and antibiotic therapy for DFI, episodes with low NRS-Scores on admission (1 to 3 points) had fewer clinical failures after therapy than those with high NRS-Scores (4-5 points). Furthermore, receiving professional nutritional intervention during hospitalization was statistically associated with a significantly shorter (by three days) hospital stay and a shorter duration (by four days) of antibiotic therapy. The number of surgical debridement in the operating theatre tended was also nonsignificantly lower in the group who had nutritional intervention. Independent vitamin supplementation provided by the patient or family members appeared to have no effect on outcomes.

The presence of a low serum albumin level on admission was significantly associated with an increased rate of clinical failures. However, we excluded the variable “albumin” from the multivariate analysis because a low albumin level can be a marker of inflammation or acute infection. In such situations, the serum albumin may reflect these confounding disorders, or any underlying hepatic dysfunction, rather than the chronicity of malnutrition [9]. As we have published previously, specific antibiotic-related variables (duration of systemic therapy, administration by the intravenous route) were not associated with the overall risk of clinical failure in these multifaceted DFI populations [2, 3, 10]. Our results confirm those of previous studies in other fields of medicine that have shown that a higher NRS-Score can be associated with a longer hospital stay [7]. Importantly in this regard is that we also found that providing nutritional interventions may decrease the duration of hospitalization.

Of note, our study was retrospective and can therefore only reveal associations, not proof of a causal relationship between nutrition and remission of DFI. While such an association is clinically plausible, it is possible that those patients who receive nutritional interventions are those who also are more compliant with other aspects of their DFI treatment, such as pressure off-loading or properly taking their antibiotic therapy [1, 2]. In such a case, the presence of a nutritional disorder could only be a hallmark of multiple comorbidities [8].

In contrast to our previously reported finding of an apparent lack of association between malnutrition and outcomes of closed deep infections in general orthopedic surgery [8], the association of therapeutic failures with malnutrition in the DFI patient could rather be related to (postoperative) wound healing problems than to bacterial infection *per se*. This aspect is very important in the literature. Many published data support a role of nutrition in wound closure in patients with a diabetic foot ulcer (DFU) [4, 5]. Nutrition is believed to be favorably connected in almost every facet of healing of DFUs, including immune function, glycemic control, hydroxyproline concentrations, weight management, and physical ability [4, 5]. Better nutrition is even associated with overall reduced all-cause mortality [10] among the multimorbid and frail DFU patients [11].

To cite concrete and recent examples, Hong et al. evaluated 771 geriatric Chinese patients with type 2 diabetes and DFU and associated three different geriatric scores ^12^(geriatric nutritional risk index (GNRI), prognostic nutritional index (PNI), and controlling nutritional status (CONUT)) to all-cause mortalities [10]. All scores were heavily influenced by the current insufficient nutritional status. The multivariable Cox regression revealed that a low GNRI (hazard ratio (HR) 2.0, 95% confidence interval 1.4-3.0), a low PNI (HR 2.0, 95% CI 1.3-3.2), and a high CONUT score (HR 1.5, 95% CI 1.1-2.2) were all independently associated with high all-cause mortality [10]. In Scotland, Chamberlain et al. [12] found a relation between a low BMI and the risk of amputation and/or all-cause mortality among diabetic patients. Most likely, the low BMI (<18.5 kg/m^2^) reflected current malnutrition of their patients. Similarly, they also suspected that a low serum glycated hemoglobin level could be associated with poor nutrition, malignancy, and frailty [11, 12]. Gazzaruso et al. investigated predictors of healing, DFU recurrence or persistence, amputation, and general mortality in 583 Italian diabetic patients [13]. The authors clearly demonstrated that a low BMI, which itself was associated with mal- or undernutrition, was an independent predictor of both, DFU persistence and overall death [13]. They proposed nutrition assessments and if needed enhanced nutritional support to all DFU patients [13]. Of note, all these nutritional DFU studies investigated a patient population without apparent infection. This is the main difference to our study, in which we included only infected cases and also accepted DFI episodes without DFU. Nevertheless, all our findings and those of the literature suggest the same: a positive role of enhanced nutrition in DFU healing [8–13] and, consequently, cure of infection (with or without underlying ulcers).

The main strengths of our study are the large database of over 1,000 DFI patients and the long duration of follow-up in a specialized, academic diabetic foot unit [6, 10]. The main limitations are the varied case-mix, retrospective nature of the study, arbitrary indication for requesting nutrition counselling (compared to a general nutrition counselling for every DFI patient), majority of operated patients (versus mostly conservative DFI therapies), lack of formal proof of a causal relationship between nutrition and therapeutic failures, and lack of an artificial variable “malnutrition” that may include a variety of nutritional parameters. However, we purposely excluded creating such a variable. Malnutrition is a complex term including high NRS-Scores, a history of starvation, presence of overweight, weight loss, and presumed low nutritional intake. Overall, the term “malnutrition in usual schemes” may help to identify sicker patients [8]. Thus, “malnutrition” may show interaction with other demographics, which we avoided. Finally, we are aware that the availability of several specialized nutritionists (as in our center) is not true for many other sites of care, especially not in resource-poor settings.

## 5. Conclusion

In our retrospective, single-center study, DFI cases with low NSR-Scores had a lower risk of therapeutic failure, and the presence of professional nutritional interventions was associated with improved outcomes. As we recognized the need for confirmatory studies, we have started a prospective evaluation of these issues [14] in 400 DFI and DFO patients. In these prospective-randomized trials, we plan to primarily investigate the duration of antibiotic therapy. Importantly, one secondary objective is the impact of nutrition interventions on the remission of DFI and DFO [14]. If we can show a benefit (at least in cases with dehiscent wounds), such an additional nutritional intervention could also be cost-effective in the management of DFI.

## Figures and Tables

**Table 1:** Characteristics of 1,013 patients operated on for diabetic foot infections.

	Remission	Clinical failure	
Characteristic (*n* = 1,013)	*n* = 758 (75%)	*n* = 255 (25%)	*p* value^∗^
Male sex	203 (80%)	591 (78%)	0.58
Median age	65 years	68 years	0.09
Insulin therapy	561 (74%)	197 (75%)	0.69
Diabetic foot osteomyelitis present	213 (84%)	669 (88%)	0.06
Undergoing renal dialysis	63 (8%)	22 (9%)	0.88
Congestive heart failure	174 (23%)	83 (33%)	**0.01**
Moderate to severe limb ischemia	559 (74%)	205 (80%)	**0.03**
Active tobacco smoker	445 (59%)	162 (64%)	**0.17**
Number of surgical debridement (median)	1	1	**0.01**
Duration of antibiotic therapy (median)	20 days	30 days	**0.01**
Parenteral route of therapy (median)	4 days	7 days	**0.01**
*Nutritional assessments on admission*			
Median NRS-Score	2 points	3 points	0.82
Median weight	87 kg	86 kg	0.75
Median body mass index	28.7 kg/m^2^	29.5 kg/m^2^	0.46
Reported weight loss	54 (7%)	20 (8%)	0.70
Median weight loss in the last 2 months	5 kg	5 kg	0.84
Median serum albumin level^+^	39 mg/L	31 mg/L	**0.02**
Regular alcohol consumption	173 (23%)	70 (27%)	0.13
*Nutrition interventions during hospitalization*			
At least one nutritionist's counselling	142 (19%)	49 (19%)	0.87
Overall number of nutritionists' interventions	1	1	0.80
Supplementation with vitamins	190 (25%)	61 (24%)	0.71

^∗^Pearson *χ*^2^ test or Wilcoxon rank sum tests. Significant results (*p* < 0.05) are *in bold*. ^+^ *= more likely to be influenced by the presence of infection rather than the nutrition level*; NRS = Nutritional Risk Screening Score 2002.

**Table 2:** Univariate and multivariate associations (*Cox regression analyses with hazard ratios and 95% confidence intervals*) targeted to the outcome “*clinical failure*”.

Clinical failures, *n* = 255	Univariate	Multivariate
Receiving insulin therapy	0.9, 0.7-1.2	0.8, 0.2-2.8
Diabetic foot osteomyelitis present	1.1, 0.8-1.5	1.1, 0.2-4.8
Peripheral arterial disease present	1.1, 0.8-1.5	-
Underwent revascularisation	1.2, 0.9-1.5	2.0, 0.6-7.2
Body mass index at admission	1.0, 1.0-1.0	0.9, 0.9-1.0
Regular alcohol consumption	1.3, 0.9-1.7	-
Smoking	1.3, 1.0-1.7	-
History of involuntary weight loss	**1.6, 1.1-2.6**	-
Amount of patient-recalled weight loss	1.0, 0.8-1.2	0.9, 0.1-6.1
Serum albumin level at admission	**0.9, 0.8-0.9**	-
NRS-Score at admission (continuous variable)	1.0, 0.7-1.3	-
NRS-Score 1 point	**0.2, 0.1-0.8**	**0.1, 0.1-0.7**
Score 2 points	**0.1, 0.1-0.5**	**0.1, 0.1-0.8**
Score 3 points	**0.2, 0.1-0.7**	**0.1, 0.1-0.7**
Score 4 points	0.2, 0.1-1.1	0.2, 0.1-2.6
Score 5 points	0.3, 0.1-1.1	0.2, 0.1-1.4
Vitamin supplementation	1.2, 0.9-1.6	1.4, 0.4-4.6
Duration of antibiotic therapy	1.0, 1.0-1.0	-
Duration of intravenous administration	1.0, 1.0-1.0	-

^∗^Significant results are displayed *in bold*. “-” = not included in the model due to interaction (effect modification) or clinical irrelevance regarding the study question; NRS = Nutritional Risk Screening Score 2002.

**Table 3:** Secondary outcomes of treatment of diabetic foot infections stratified by whether or not there was accompanying nutritionist intervention.

Secondary outcome	Nutrition intervention	*pvalue*	No nutrition intervention
Median length of hospital stay	14 days	0.02	17 days
Median number of surgical debridement	1	0.08	1
Median duration of antibiotic therapy	18 days	0.01	22 days

## Data Availability

We may share anonymous key data upon reasonable scientific request to the corresponding author.
